# Delimiting 33 *Carpinus* (Betulaceae) species with a further phylogenetic inference

**DOI:** 10.1093/aobpla/plac006

**Published:** 2022-02-21

**Authors:** Congcong Dong, Zhiqiang Lu, Han Zhang, Jianquan Liu, Minjie Li

**Affiliations:** State Key Laboratory of Grassland Agro-ecosystems, College of Ecology, Lanzhou University, Lanzhou 730000, People’s Republic of China; CAS Key Laboratory of Tropical Forest Ecology, Xishuangbanna Tropical Botanical Garden, Chinese Academy of Sciences, Mengla 666303, Yunnan, People’s Republic of China; State Key Laboratory of Grassland Agro-ecosystems, College of Ecology, Lanzhou University, Lanzhou 730000, People’s Republic of China; State Key Laboratory of Grassland Agro-ecosystems, College of Ecology, Lanzhou University, Lanzhou 730000, People’s Republic of China; Key Laboratory of Bio-Resource and Eco-Environment of Ministry of Education, College of Life Sciences, Sichuan University, Chengdu 610065, People’s Republic of China; State Key Laboratory of Grassland Agro-ecosystems, College of Ecology, Lanzhou University, Lanzhou 730000, People’s Republic of China

**Keywords:** *Carpinus*, ITS sequences, Phylogeny, Species delimitation

## Abstract

*Carpinus* (Betulaceae) has approximately 52 species distributed in the Northern Hemisphere, with many species of *Carpinus* found in China. However, the species boundaries and phylogenetic relationships remain poorly understood. This study reported ITS sequences for 225 individuals of 33 *Carpinus* species, mainly from China. We also included eight *Ostrya* species in our analyses, the closely related sister group of *Carpinus*. We aimed to delimit these species based on ITS sequences and clarify their phylogenetic relationships by constructing tree-like topology and networks at population level. We found that only 17 of 33 species could be delimited from the closely related ones based on species-specific mutations in ITS sequence variation, including all species of sect. *Distegocarpus*, and sect. *Carpinus* subsect*. Carpinus. Carpinus* subsect*. Carpinus* contained two endangered species, although one seemed to be a recently originated allopolyploid species with genetic additivity from two likely parents in the ITS sequence variation. Sixteen species of sect. *Carpinus* subsect. *Polyneurae* were classified into three species complexes, in each of which two or more could be not distinguished from each other. The closely related species of these complexes may still diverge at the early stage without genetic distinction in the nuclear ITS sequences because of too short of divergence time and frequent gene flow. Otherwise, some species may be established based on the intraspecific variations without genetic bases for an independently evolving unit.

## Teaser

By using nuclear ITS sequence of the genus *Carpinus*and its sister genus sampled from its distribution centre in China, a phylogenetic analysis was performed as a test of species delimitation, aiming to providing accurate species boundaries and phylogenetic relationships for conservation or other aims. Our results showed that 17 of 33 species could be delimited from the closely related ones, while the remaining 16 species were classified into three species complexes because of too short a divergence time and frequent gene flow.

## Introduction

Accurate species delimitation is critical in most biological disciplines because species generally is the basic element in many studies (Simpson 1951; Mayr 1982). For example, such a failure of accuracy may result in over- or underestimation of the total number of the endangered species for conservation aims (Richardson 1978; Mayden 1999). However, defining a species and delimiting species boundaries remains highly disputed (Mayden 1997; De Queiroz 2007; Liu 2016). Except for morphological gaps at the population level, numerous studies suggest that genetic distance at the nuclear orthologous genes or chloroplast (cp) DNAs should be used to delimit species (Yang and Rannala 2010; Hu *et al.* 2015; Liu 2016; Feng *et al.* 2021), which can be further referenced as a molecular marker to barcode and identify species (Besansky *et al.* 2003; Kress *et al.* 2005; Shaw *et al.* 2005). Several cpDNAs, such as rbcL and matK, trnH-psbA and trnL-F, were first suggested to barcode and delimit plant species (Besansky *et al.* 2003; Shaw *et al.* 2005; Kress and Erickson 2007; CBOL Plant Working Group 2009). The cpDNAs are uniparentally (mostly maternally) inherited without discernible recombination. Therefore, besides incomplete lineage sorting and low divergence, these cpDNA markers may fail to distinguish the closely related species because of hybridization and organelle introgression (Degnan and Rosenberg 2009; Liu 2016; Chan *et al.* 2017). The effectiveness of ITS sequence variation has been widely tested in delimiting species in diverse groups of plants (e.g. Wang *et al.* 2011; Hu *et al.* 2015; Feng *et al.* 2021), although it still fails to distinguish two species with an extremely short divergence.

This study aimed to delimit the genus *Carpinus* (Betulaceae) using ITS sequences. This genus contains approximately 52 species, disjunctively distributed in Europe, North America and East Asia as a typical monoecious genus with both pollen and fruits dispersed by the wind over long distances (Li and Skvortsov 1999). Approximately 35 species were found in East Asia, and 29 were endemic to China (Li and Skvortsov 1999; Fu 2000). Two sections (sects.) *Distegocarpus* and *Carpinus*, and three subsections (subsects. *Carpinus*, *Monbeigianae* and *Polyneurae*) have been recognized within *Carpinus* (Li and Cheng 1979). The delimitation between subsects. *Monbeigianae* and *Polyneurae* was distinct based on statistical analyses of morphological characters from leaves and fruit bracts (Jeong and Chang 1997). Congruently, intraspecific variations and morphological polymorphisms led to significant difficulties in delimiting some close *Carpinus* species (Hu 1964; Li and Cheng 1979; Li and Skvortsov 1999; Yoo and Wen 2007; Li 2008). During the phylogenetic construction of *Carpinus*, the ITS sequences were reported for 13 species with only nine different sequences identified (Yoo and Wen 2007), suggesting that at least four species shared the same ITS sequence variation with others. Similarly, the combined cpDNAs (*matK*, *trnL-trnF* and *psbA-trnH*), of which at least two (*trnL-trnF* and *psbA-trnH*) were used to discriminate the closely related species (Kress and Erickson 2007; CBOL Plant Working Group 2009), showed the very low variation between sampled species. Only five different sequences were identified, with at least eight species sharing the same cpDNA sequence variations with other species (Yoo and Wen 2007). In several studies, new species were described for the genus *Carpinus* (Liu and Lin 1986; Liang and Zhao 1991; Yi 1992; Tong *et al.* 2014; Lu 2017; Lu *et al.* 2018), and totalling about 40 Chinese *Carpinus* species have been recorded, among which, two prominently endangered species were classified as *C. tientaiensis* and *C. putoensis* (Qin and Zhao 2017). It was necessary to scale interspecific genetic distinctness between these species using an ITS marker that designated the sister and closely related species in numerous groups (Li *et al.* 2011; Wang *et al.* 2011; Hu *et al.* 2015; Feng *et al.* 2021). It remains unknown how many, especially the two endangered tree species, are ‘good’ species with evolutionary distinctness as independently evolving units.

This study collected 191 individuals of 22 species for the genus *Carpinus.* Multiple individuals and populations were collected for most species. We downloaded all available ITS sequences for *Carpinus* and then *Ostrya*, a sister group of *Carpinus* in Betulaceae. We aimed to examine whether ITS sequences showed variation between different individuals and populations of each species. We further explored whether these sequences could distinguish most species of the genus defined by morphological traits found in most plant groups (Li *et al.* 2011). In addition, we also hoped to explore whether such a delimitation showed variation range of a species throughout all populations. Finally, we used the ITS sequences to construct phylogenetic relationships for this genus. These results are beneficial for improving our understanding of taxonomy, species divergence and phylogeny of the genus.

## Materials and Methods

### Species sampling, sequencing and alignment

According to the Flora of China (33 species; Li and Skvortsov 1999) and the recently announced new species of *Carpinus*, 191 samples of 22 *Carpinus* species were collected with more than two samples included for each species (see detail in **Supporting Information—Table S1**). The detailed information of collection sites for these samples is shown in **Supporting Information—Table S1**. All fresh leaves of the samples were dried in silica gel for total DNA extraction. The specimens of these samples were deposited in the Herbarium of Lanzhou University, China. We also downloaded 34 ITS sequences of 11 *Carpinus* species, 27 ITS sequences of eight *Ostrya* species and two ITS sequences of two *Corylus* species from NCBI with the accession numbers listed in **Supporting Information—Table S2** for further experimentation.

The total genomic DNA (gDNA) for each target sample was extracted according to the modified CTAB method, with approximately 30 milligrams (mg) of dried leaves used (Li *et al.* 2013). The PCR amplification mixture had a total volume of 25 microlitres (μL), made up of 10–40 nanograms per microlitre (ng μL^−1^) of plant gDNA, 2.5 μL of 10× PCR buffer, 0.5 millimoles per litre (mmol L^−1^) of dNTPs, 2 mμL L^−1^ of ITS forward and reverse sequence primers (Wen and Zimmer 1996), 0.2–0.3 μL rTaq enzyme [5 units per microlitre (U μL^−1^); Takara, Dalian, China] and ddH_2_O. The PCR reaction was performed on a T1 PCR instrument (Biometra, Göttingen, Germany) using an optimized program in which the initial denaturation temperature was at 94 °C for 4 minutes (min), then 36 cycles at 94 °C for 45 seconds (s), 54 °C renaturations for 50 s, 72 °C extensions for 90 s and one 72 °C extension for 10 min after the end of the 36th cycle. All PCR products were detected by 1% agarose gel electrophoresis, and then the products were purified using the TIAN quick Midi Purification Kit according to the protocols (Beijing, China). All purified samples were sequenced using Sanger technology in Tsingke (Wuhan, China). The newly generated ITS sequences were stored in NCBI with the accession number MW928890–MW929080 and OK560470–OK560480 (see detail in **Supporting Information—Table S2**).

We found that the ITS sequence (GenBank accession no. AF432051) of the prominently endangered species *C. putosensis* was clustered together with *C. mianningensis* (see the Results section). Given the chromosome number of *C. putoensis* with 2n = 14x = 112 (Meng *et al.* 2004), we phased the ITS sequences of *C. putoensis* to ascertain whether it is an allopolyploid or autopolyploid according to the following procedures. The purified PCR products of *C. putosensis* were recombined into pMD 19-T vectors according to the protocols of this vector kit (Baosheng, Dalian, China). The reaction system was prepared in 15 μL with 1.5 μL pMD 19-T, 1.5 μL of PCR product, 4.5 μL aseptic water (H_2_O) and 7.5 μL of ligation solution. The reaction was carried out in MultiTime III circulating water bath system (America, GE) at 16 °C for 1 h. The recombinant pMD 19-T vectors were then transformed into DH5α competent Cell (Dongsheng Biotech), and blue–white screening was used to select the positive clones for Sanger sequencing in Tsingke (Wuhan, China) by the *Bca*BEST™ sequencing primers (Baosheng, Dalian, China).

We employed SeqMan (DNAstar; Burland 2000) to edit the forward and reverse sequences to obtain a consensus sequence for each newly generated ITS sequence. All sequences were aligned using MEGA v7.0 (Kumar *et al.* 2016), employing fine manual adjustment. In the aligned sequence data set, insertions and deletions were regarded as missing data in phylogenetic analyses.

### Phylogenetic analyses

Bayesian inference (BI), maximum likelihood (ML) and maximum parsimony (MP) approaches were deployed to reconstruct the phylogeny of *Carpinus* based on the ITS data set. The Akaike information criterion (AIC) was used to evaluate the best-fitting model in jModelTest v2.1.7 (Darriba *et al.* 2012). The ML analysis was implemented using RAxML version 8.2.8 (Stamatakis 2014) with 1000 bootstrap replicates using the GTRGAMMA nucleotide substitution model estimated from AIC. Bayesian inference analysis was performed in MrBayes v3.2.0 (Ronquist and Huelsenbeck 2003) from a random starting tree with the best-fitting GTR model, and then four Markov Chain Monte Carlo (MCMC) chains consisting of three heated and one cold chain were run to estimate the posterior distribution of the model parameters. We ran 10 000 000 generations for the trees and drew a tree every 1000 generations. The first 20 % of trees were discarded as burn-in, and the remaining trees were applied to infer the majority-consensus tree and the posterior probabilities. Maximum parsimony analysis was performed using PAUP v4.0 (Swofford 2002). Three analyses were carried out independently using four MCMC (three hot and one cold chain), with 10 000 000 generations for each run and sampling every 1000 generations. The first 25 % of trees were discarded as burn-in, and the remaining trees were used to construct a 50 % majority-rule consensus tree. FigTree version 1.4.4 (Rambaut 2018) visualized the final trees.

The neighbour-net graphic was analysed in SplitsTree4 (Hudson and Bryant 2006) using an uncorrected *P*-distance method with 1000 replicates for bootstrapping to support and further ensure the phylogenetic relationships. DnaSP v5.10.01 (Librado and Rozas 2009) generated ribotypes among *Carpinus* species. Genealogical relationships of ribotypes were inferred from a median-joining method in Network 4.6.1.3 (Bandelt *et al.* 1999). The geographical distribution of ribotypes was recorded at a species-defined population level.

## Results

### Sequence sampling and variation

In this study, we downloaded two *Corylus* species as outgroups (*C. california* and *C. heterophylla*) and 27 ITS sequences for eight *Ostrya* species from the NCBI database, including two *O. carpinifolia*, seven *O. japonica*, two *O. knowltonii*, two *O. multinervis*, five *O. rehderiana*, four *O. trichocarpa*, three *O. virginiana* and two *O. yunnanensis***[see****Supporting Information—Table S2****]**. We also retrieved 34 ITS sequences of 11 *Carpinus* species from the NCBI database, including six *C. betulus*, five *C. caroliniana*, five *C. japonica*, two *C. kawakamii*, two *C. laxiflora*, two *C. mianningensis*, three *C. orientalis*, one *C. putoensis*, two *C. rankanensis*, three *C. tibetana* and three *C. tientaiensis***[see****Supporting Information—Table S2****]**. We newly generated 202 ITS sequences of 23 *Carpinus* species, including four *C. chuniana*, seven *C. cordata*, two *C. fangiana*, nine *C. fargesiana*, 10 *C. henryana*, 22 *C. hupeana*, five *C. langaoensis*, two *C. londoniana*, two *C. mollicoma*, two *C. monbeigiana*, two *C. omeiensis*, eight *C. polyneura*, six *C. pubescens*, five *C. purpurinervis*, 11 ITS clones of *C. putoensis*, two *C. rupestris*, three *C. shensiensis*, three *C. stipulate*, 15 *C. sungpanensis*, 11 *C. tsaiana*, 36 *C. tschonoskii*, three *C. turczaninowii* and 32 *C. viminea***[see****Supporting Information—Table S1****]**. This process obtained 236 ITS sequences of 33 *Carpinus*, including two European species (*C. betulus* and *C. orientalis*), one North American species (*C. caroliniana*) and 30 Asian species. In combination with 27 *Ostrya* sequences and two *Corylus* sequences, the length of these 265 ITS sequences varied between 599 base pairs (bp) to 603 bp with the alignment length of 613 bp. A total of 102 parsimony informative sites were identified among these ITS sequences.

### Phylogenetic analyses

**Figure 1.**
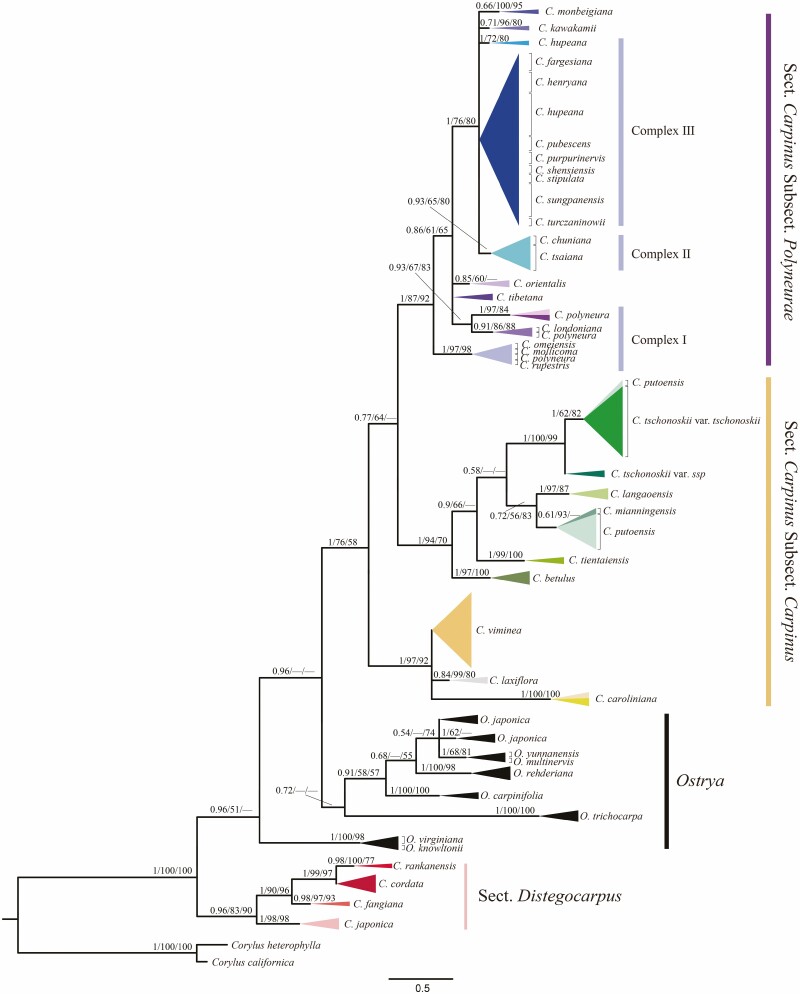
Phylogenetic analysis of *Carpinus*, with *Ostrya* and *Corylus* as the outgroup based on ITS sequences. (A) Tree-like topology. Bayesian inference (BI) phylogenetic tree is shown due to the similar topologies with maximum likelihood (ML) and maximum parsimony (MP). The numbers on the branches indicate PP/BS/MP support values from BI posterior probabilities, ML bootstrap supports and MP bootstrap values. — indicates PP/BS/MP values less than 0.5/50/50. The infrageneric categories of *Carpinus* are represented by different colours. I, II and III represent the three species complexes in subsect. *Polyneurae*.

The resulting trees showed an almost identical tree topology among BI, ML and MP analyses (Fig. 1). Our phylogenetic tree revealed that all *Carpinus* species were clustered into two groups, corresponding to the previous morphologically defined sections, i.e. sect. *Distegocarpus* (PP/BS/MP = 0.96/83/90) and sect. *Carpinus* (PP/BS/MP = 1/76/58), in which the support values successively represented as BI posterior probability (PP), ML bootstrap support (BS) and MP bootstrap value. However, *Carpinus* was considered non-monophyletic because a tentative affinity between sections *Carpinus* and *Ostrya* (PP/BS/MP = 0.96/<50/<50) was observed, where both were sister to sect. *Distegocarpus* with high support values (PP/BS/MP = 1/100/100) (Fig. 1). Within sect. *Distegocarpus*, four species were well delimited, and *C. japonica* (PP/BS/MP = 1/98/98) and *C. fangiana* (PP/BS/MP = 0.98/97/93) were successively diverged from the closest related *C. rankanensis* (PP/BS/MP = 0.98/100/77) and *C. cordata* (PP/BS/MP = <0.5/<50/<50). However, just 13 of the 29 sect. *Carpinus* species were discriminated from other closely related species. In addition, three highly supported clades were identified in the sect. *Carpinus* and species delimitation in the first two clades was well solved but not in the third. We found that *C. caroliniana* (PP/BS/MP = 1/100/100), *C. laxiflora* (PP/BS/MP = 0.84/99/80) and *C. viminea* (samples were paraphyletic) joined in the first clade (PP/BS/MP = 1/97/92) with unresolved interspecific relationships (Fig. 1). Similarly, species were successfully divided into monophyletic lineages in the second clade, except the ITS clones of *C. putoensis*, which clustered together with *C. mianningensis* (PP/BS/MP = 0.61/93/<50) and *C. tschonoskii* var. *tschonoskii*, respectively (PP/BS/MP =1/62/82; Fig. 1). Further, we found that the European *C. betulus* (PP/BS/MP = 1/97/100) and Asian *C. tientaiensis* (PP/BS/MP = 1/99/100) were successfully sister to other Asian species (PP/BS/MP = 1/94/70 and PP/BS/MP = 0.9/66/<50). *Carpinus putoensis*–*C. mianningensis* showed a genetic affinity (PP/BS/MP = 0.72/56/83) with *C. langaoensis* (PP/BS/MP = 1/97/87), and all of them were sister to *C. putoensi* - *C. tschonoskii* (PP/BS/MP = 1/100/99) with low support values (PP/BS/MP = 0.58/<50/<50). Contrary to the first two clades, only four of the 20 species in the third clade (PP/BS/MP = 1/87/92) were identified from the closely related other, including *C. tibetana* (samples were paraphyletic), *C. orientalis* (PP/BS/MP = 0.85/60/<50), *C. monbeigiana* (PP/BS/MP = 0.66/100/95) and *C. kawakamii* (PP/BS/MP = 0.71/96/80). The rest of the species were clustered into three species complexes. *Carpinus londoniana*, *C. mollicoma*, *C. omeiensis*, *C. polyneura* and *C. rupestris* formed the basal species complex I; *C. chuniana* and *C. tsaiana* constituted species complex II and were sister to species complex III, including *C. hupeana*, *C. fargesiana*, *C. henryana*, *C. pubescens*, *C. purpurinervis*, *C. shensiensis*, *C. stipulata*, *C. sungpanensis* and *C. turczaninowii*.

**Figure 2.**
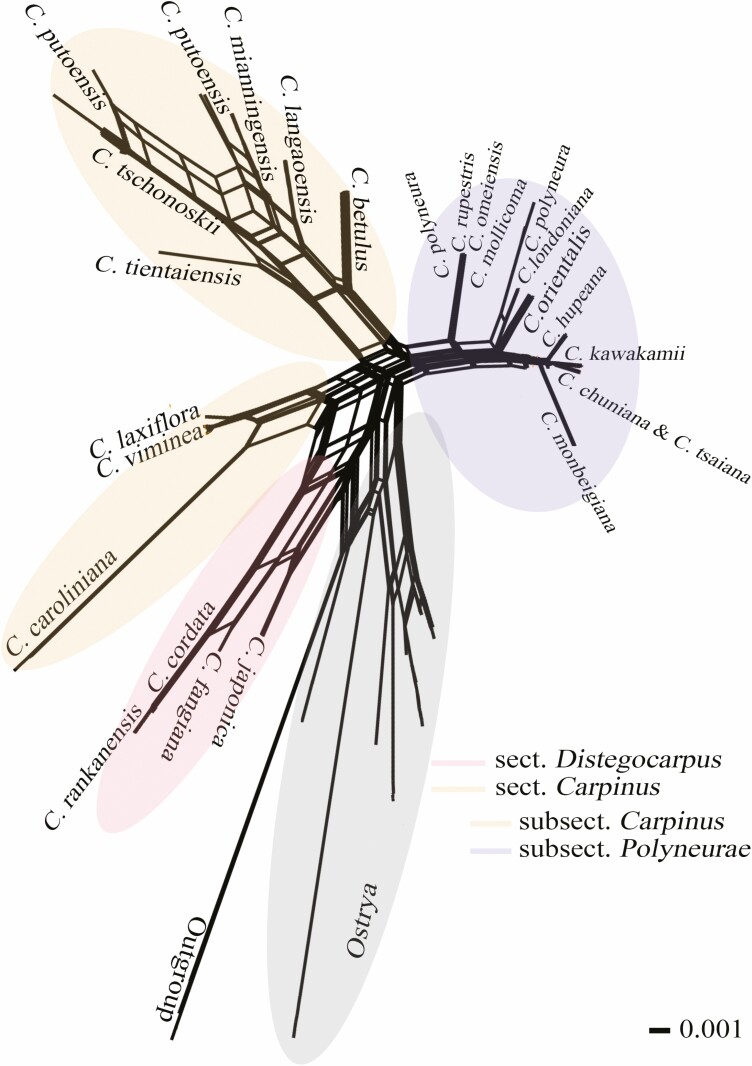
Neighbour-net graph of *Carpinus*, *Ostrya* and *Corylus* using ITS sequences. The infrageneric categories of *Carpinus* correspond to those in Fig. 1. Within in subsect. *Polyneurae*, members of the first two species complexes are shown, but not for the members of species complex III due to the narrowly meshed networks among those species, except some individuals of *C. hupeana*.

We inferred the neighbour-net graph using uncorrected *P*-distance to confirm species boundaries and phylogenetic affinities further. Compared to the tree-like topology, we found a clear genetic split between the genera *Carpinus* and *Ostrya*. The sects. *Distegocarpus* and *Carpinus* formed a separate group and were sisters (Fig. 2). However, species delimitation and interspecific relationships revealed by the neighbour-net graph were principally consistent with the ITS tree topology (Figs 1 and 2). Within sect. *Distegocarpus*, the four species involved were well delimited. Within the first clade of sect. *Carpinus*, a closer relatedness between *C. laxiflora* and *C. viminea* was supported, and both were sisters to *C. caroliniana*. The interspecific relationships among well-clustered species in the second clade agreed with those revealed by the tree-like topology (Figs 1 and 2). Like the tree-like topology, species falling into the third clade were difficult to delimitate and mostly linked with the narrowly meshed networks (Fig. 2). These species were further clustered into three species complexes, and their relationships were congruent with the tree-like topology (Fig. 1).

### Ribotype network of *Carpinus* species

**Figure 3.**
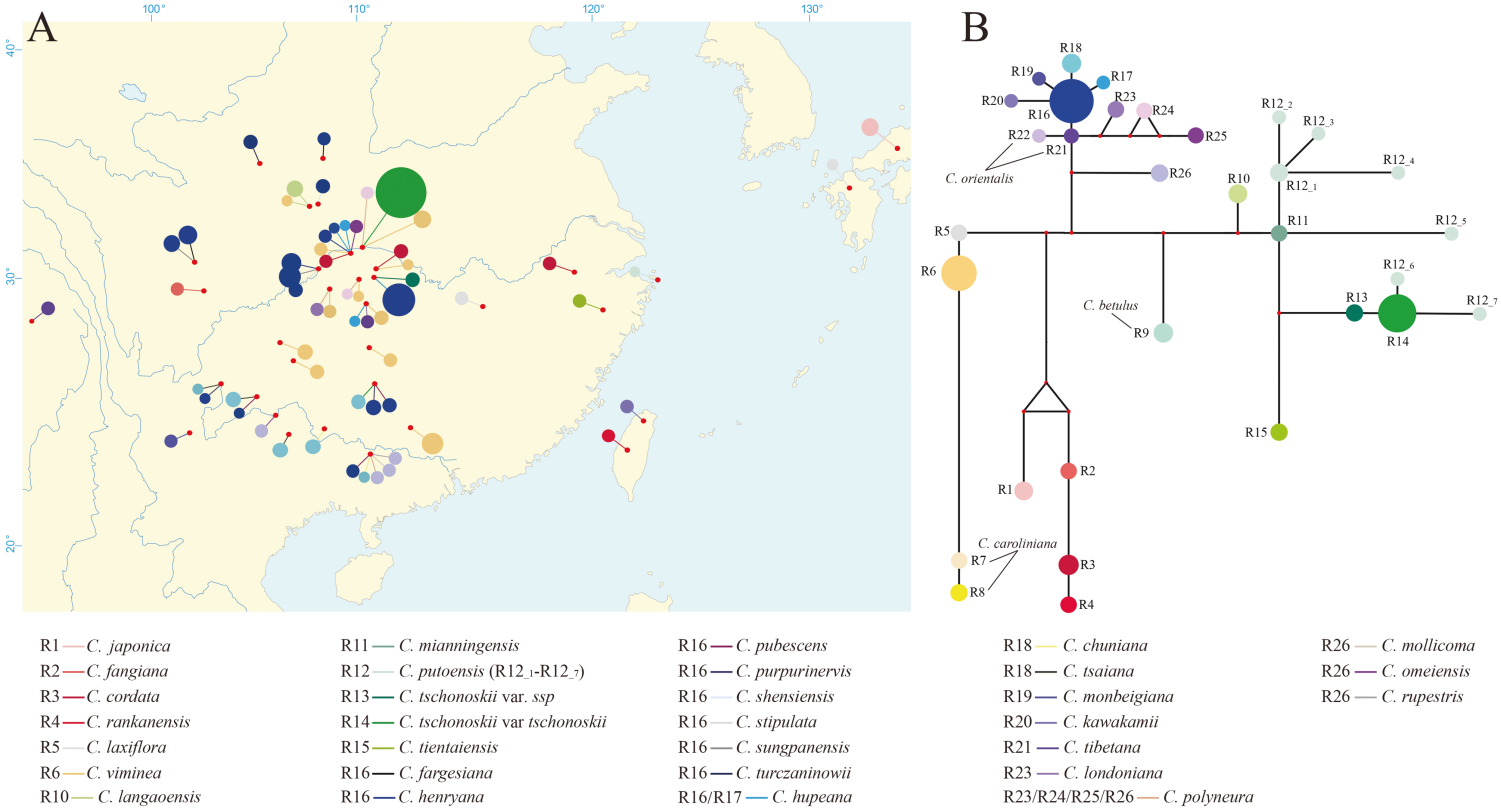
Ribotype analysis of *Carpinus* species based on ITS sequences. (A) Geographical distribution of ribotypes of Asian *Carpinus* species. The solid lines indicate the corresponding relationships between species and ribotypes. (B) Ribotype network of *Carpinus* species. Circle size indicates the ribotype frequency. The smallest circles indicate extinct or unsampled ribotypes.

We reconstructed the ribotype network among the *Carpinus* species to examine interspecific relationships (Fig. 3B). The alignment length of the 236 ITS sequences of 33 species of *Carpinus* was 605 bp. According to the 69 parsimony informative sites, a total of 26 ribotypes (R1–R26) were detected for 33 population-defined species **[see****Supporting Information—Table S3****]**, when all ribotypes (R12__1_–R12__7_) representing different ITS clones of *C. putoensis* were treated as R12. We found that 19 (R1–R15 and R19–R22) of 26 ribotypes were species-specific and capable of delimiting 17 species from each other. Consistent with the tree-like topology and neighbour-net graph, two sections of *Carpinus* were split in the network. Within sect. *Distegocarpus*, four species-specific ribotypes (R1–R4) were identified, and the ribotype connections corresponded to the interspecific relationships recovered by the tree-like topology and neighbour-net graph (Figs 2 and 3B). Within the sect. *Carpinus*, three clades were discerned, and the species-specific ribotypes (R5–R15) were identified in the first two clades, while the interspecies-shared ribotypes were primarily detected in the third clade (e.g. R16, R18 and R26). This result was indicative of the three species complexes. In detail, four ribotypes (R5–R8) were identified for the three species of the first clade, and two ribotypes were observed in *C. caroliniana* (R7–R8) (Fig. 3). Seven ribotypes (R9–R15) were detected for the six species of the second clade, and two ribotypes (R13–R14) were detected in *C. tschonoskii* (Fig. 3). Although *C. putoensis* fell together with *C. mianningensis* and *C. tschonoskii* in the tree-like topology and neighbour-net graph (Figs 1 and 2), the species-specific ribotypes were identified for *C. putoensis* (R12__1_–R12__7_). Within the species-specific ribotypes for *C. putoensis* (R12__1_–R12__7_), two were linked to R14 of *C. tschonoskii*, and the remaining five were connected with R11 of *C. mianningensis* (Fig. 3B). In the third clade, a ‘star-like’ network was observed, and the dominant R16 was placed at the centre position. Furthermore, we found that R16 was mainly shared by species complex III, including *C. fargesiana*, *C. henryana*, *C. hupeana*, *C. pubescens*, *C. purpurinervis*, *C. shensiensis*, *C. stipulata*, *C. sungpanensis* and *C. turczaninowi*. R18 was shared by *C. chuniana* and *C. tsaiana* within species complex II, and R26 was observed among *C. mollicoma*, *C. omeiensis*, *C. polyneura* and *C. rupestris* belonging to the species complex I (Fig. 3B). In addition, R21 was shared by *C. tibetana* and *C. orientalis*, and R23 was shared by *C. londoniana* and *C. polyneura*. *Carpinus hupeana* (R16–R17) and *C. orientalis* (R22–R23) were found to have two ribotypes, respectively. Multiple ribotypes (R23–R26) were identified in *C. polyneura*. A species-unique ribotype was also observed in *C. monbeigiana* (R19) and *C. kawakamii* (R20), respectively **[see****Supporting Information—Table S3****]**.

When the identified ribotypes were projected to the geographical map at species-defined populations, we found that the species both of sect. *Distegocarpus* and subsect. *Carpus* in the phylogenetic analyses showed no shared ribotypes with the the North American and Euro species even when they were distributed at the same localities (Fig. 3A). However, the interspecies-shared R16 was widespread in the whole distribution range and identified among different species collected in the same localities. The shared R26 was found in different species (e.g. *C. mollicoma*, *C. polyneura* and *C. rupestris*) sampled at the same locality and also found in a distinct locality where *C. omeiensis* was sampled. The shared R18 was observed in different localities for different species. Noticeably, R18 and R26 were mainly distributed in the south-east inland of China, specifically occurring at localities with closer geographical distances. The shared R21 occurred all across Eurasia.

## Discussion

It is critical to determine whether one described species represents an independently evolving unit because some species are established based on intraspecific variants or interspecific hybrids (Liu 2016). The establishment of independently evolving units is crucial for conserving endangered species (Wiens 2007). In this study, we used the widely used genetic marker ITS for species identification (Li *et al.* 2011) to examine species distinctness of the genus *Carpinus*, containing two well-known endangered tree species (Qin and Zhao 2017). By sampling multiple individuals for each species, we aimed to examine whether a genetic gap could exist between two species to exclude intraspecific variations and interspecific hybrids at the utmost extent. We clustered all exampled individuals into three groups, sect. *Distegocarpus*, subsect*. Carpinus* and subsect. *Polyneurae*. We found that only 17 of 33 species seemed to be ‘good’ species, whose ITS sequence variations could diagnose. In addition, most species within the sect. *Distegocarpus*, and subsect*. Carpinus* were well delimited. However, some species of subsect. *Polyneurae* could not be discriminated by the ITS fragment from the closely related sister species. Recent divergence and frequent hybridization may have blurred interspecific boundaries in these species complexes, although further population-level statistical analyses are needed to examine whether morphological gaps exist between these ‘species’ without genetic distinctness.

### ‘Good’ species in two of three phylogenetic groups

The phylogeny-based method distinguished species because all individuals of one species were assumed to be derived from one common ancestor and therefore clustered together as one monophyletic group (Liu 2016). In addition, such analyses can further construct phylogenetic relationships. Phylogenetic analyses of 236 ITS sequences from 33 *Carpinus* species revealed two distinct clades corresponding to the morphology-related sects. *Distegocarpus* and *Carpinus* (Li and Skvortsov 1999). This result is consistent with the previous phylogenetic results based on ITS and the nuclear low-copy nitrate reductase (*Nia*) gene (Chen *et al.* 1999; Kato *et al.* 1999; Yoo and Wen 2007; Li 2008; Grimm and Renner 2013; Lu *et al.* 2018).

No shared ribotypes were observed between sect. *Distegocarpus* and sect. *Carpinus*, suggesting two independent evolutionary lineages without gene flow (Fig. 3B). Four species in sect. *Distegocarpus* were delimited as independently evolving units (Fig. 3B). Within sect. *Carpinus*, three groups were identified but did not correspond to the three subsections (Figs 1, 2 and 3B). According to bract characters (Li and Skvortsov 1999), the first two groups belong to subsect. *Carpinus* whose bracts were lobed at bases of inner and outer margins, while the third one corresponded to subsects. *Monbeigianae* and *Polyneuae* with only an inflexed lobe or auricle at the base of the inner margins. This result suggests that these two subsections could be incorporated into one, i.e. subsect. *Polyneurae* (Jeong and Chang 1997) (Fig. 1). In addition, no ribotype was shared between nine species of subsect. *Carpinus*, of which, eight species including *C. betulus*, *C. caroliniana*, *C. mianningensis*, *C. langaoensis*, *C. laxiflora*, *C. tientaiensis*, *C. tschonoskii* and *C. viminea* were well discriminated (Fig. 3B), with an exception that the obtained ITS clones of *C. putoensis* were mixed with *C. mianningensis* and *C. tschonoskii* (Figs 1 and 2).

It should be noted that *C. tientaiensis* has a very small population size, with only 19 wild individuals recorded (Qin and Zhao 2017). The ITS sequence of *C. tientaiensis* differs from those of the closely related species with four species-specific mutations. Therefore, *C. tientaiensis* is a well-delimited ‘good’ species. The field investigations showed that *C. putoensis* is a critically endangered species, containing one individual that was more than 250 years old, occurring only on the Zhoushan Islands of Zhejiang Province, China (http://www.iplant.cn/rep/prot/Carpinus%20putoensis). The chromosome number of *C. putoensis* was found to be a polyploid species with 2n = 14x = 112 (Meng *et al.* 2004). Our phased ITS sequences from *C. putoensis* suggested that one type of ITS from this species was derived from *C. mianningensis*, and other ITS sequences were derived from *C. tschonoskii*. Therefore, *C. putoensis* may originate from the hybrid polyploidization between *C. mianningensis* and *C. tschonoskii.* Therefore, *C. putoensis* remains an independently evolving lineage because the allopolyploidization immediately leads to direct postzygotic isolation (Li *et al.* 2021). However, because of the small population (one individual), rare sexual recombination, tree life longevity and early speciation stage, multiple ITS copies from both parents have not experienced the concerted evolution into a single sequence and not accumulated the species-specific mutations.

### Low species discrimination in subsect. *Polyneurae*

There are more described species (20) in the subsect. *Polyneurae* than the other two groups: sect. *Distegocarpus* (4) and subsect*. Carpinus* (9). In addition, subsect. *Polyneurae* has a large distributional range than sect. *Distegocarpus* and subsect*. Carpinus* (i.e. the distribution of R16; Fig. 2). Most species of subsect. *Polyneurae* have large populations, and none of them have been listed as endangered. Surprisingly, only 4 out of 20 species were distinguished (i.e. *C. kawakamii*, *C. monbeigiana*, *C. orientalis* and *C. tibetana*). However, the rest of the 16 species were clustered into three different species complexes due to the shared ribotypes within each complex. These species complexes can be distinguished from others and contain at least three ‘good’ species. Although *C. londoniana* has similar bracts with *C. tientaiensis* and *C. viminea* of subsect*. Carpinus*, *C. londoniana* was clustered with species of subsect. *Polyneurae* and shared R23 with *C. polyneura* in phylogenetic analyses (Figs 1 and 2). Species complex I comprised *C. londoniana*, *C. mollicoma*, *C. omeiensis*, *C. polyneura*, and *C. rupestris*, with only the shared R26 detected and the rest of the ribotypes (R23–R25) mostly unique to *C. polyneura* (Fig. 3B). Species complex II comprised *C. chuniana* and *C. tsaiana*, where R18 was detected between them (Fig. 3B). Compared to the first two, species complex III comprised nine species, with only the widespread R16 observed among these species, with R17 specific to *C. hupeana* (Fig. 3B).

Three explanations may account for low species discrimination in the three species complexes of subsect. *Polyneurae.* Firstly, species divergence within these complexes is still at the early stage of evolution. Although morphological distinctness can be detected, unique and specific mutations have not accumulated between these closely related species. Secondly, the described species within some species complexes (e.g. *C. mollicoma*, *C. polyneura*, and *C. rupestris*) are adjacently distributed. These adjacent distributions promote frequent gene flow through wind-mediated pollen and fruit dispersal (Li and Skvortsov 1999) but substantially reduce divergence for evolving units. The occurrence records suggest that most of these described species were distributed widely with large population sizes. The large population sizes and the long generation times of these tree species may further delay the speciation process and shorten the time scales for producing and accumulating unique mutations because of fewer intraspecific recombination and frequent interspecific gene flow (Li and Skvortsov 1999). However, the endangered species with small population sizes (e.g. *C. tientaiensis*) may accumulate specific mutations quickly because of the increased intraspecific recombination and reduced hybridization with the closely related species. Finally, it needs to be noted that some species in these species complexes are described by intraspecific variations rather than independently evolving units (Liu 2016). Within this scenario, morphological and genetic gaps could not be distinguished through population-level analyses even when based on sensitive molecular markers for species divergence, such as genome re-sequencing data (e.g. Li *et al.* 2018; Yang *et al.* 2019).

In the future, related studies should be conducted to confirm retaining or incorporating these species because the different taxonomic species should represent independently evolving lineages with both morphological and genetic gaps at the population level despite the early speciation stages (Liu 2016).

## Supporting Information

The following additional information is available in the online version of this article—

Table S1. The collection information of 191 individuals in Carpinus and the haplotypes of all individuals in this study.

Table S2. All sequences downloaded from NCBI in this study.

Table S3. The haplotypes of all Carpinus samples in this study and their corresponding species.

plac006_suppl_Supplementary_Tables

## Data Availability

The newly generated data were submitted in NCBI (https://www.ncbi.nlm.nih.gov/) with the GenBank accession numbers shown in **Supporting Information—Table S1**; the GenBank accession numbers of downloaded sequences from NCBI are shown in **Supporting Information—Table S2**.

## Notes

**Evolution & Diversity. Chief Editor**: Jeremy Beaulieu

## Appendix

Forty species and eight varietas in the genus *Carpinus* in China and the infrageneric categories based on a combination of genetic and morphological differences:

1. Sect. *Distegocarpus*
1.1. *Carpinus cordata*
1.1.a. *Carpinus cordata* var. *cordata*
1.1.b. *Carpinus cordata* var. *mollis*
1.1.c. *Carpinus cordata* var. *chinensis*
1.2. *Carpinus fangiana*
1.3. *Carpinus rankanensis*
1.3.a. *Carpinus rankanensis* var. *rankanensis*
1.3.b. *Carpinus rankanensis* var. *matsudae*
2. Sect. *Carpinus*—*Carpinus* sect. *Eucarpinus*
2.1. Subsect. *Carpinus*
2.1.1. *Carpinus langaoensis*
2.1.2. *Carpinus laxiflora*
2.1.3. *Carpinus londoniana*
2.1.3.a. *Carpinus londoniana* var. *londoniana*
2.1.3.b. *Carpinus londoniana* var. *xiphobracteata*
2.1.3.c. *Carpinus londoniana* var. *lanceolata*
2.1.3.d. *Carpinus londoniana* var. *latifolia*
2.1.4. *Carpinus mianningensis*
2.1.5. *Carpinus tientaiensis*
2.1.6. *Carpinus tschonoskii*
2.1.6.a. *Carpinus tschonoskii* var. *tschonoskii*
2.1.6.b. *Carpinus tschonoskii* var. *falcatibracteata*
2.1.7. *Carpinus viminea*
2.1.7.a. *Carpinus viminea* var. *viminea*
2.1.7.b. *Carpinus viminea* var. *chiukiangensis*
2.2. Subsect. *Polyneurae*
2.2.1. *Carpinus chuniana*
2.2.2. *Carpinus dayongina*
2.2.3. *Carpinus fargesiana*
2.2.3.a. *Carpinus fargesiana* var. *fargesiana*
2.2.3.b. *Carpinus fargesiana* var. *hwai*
2.2.4. *Carpinus firmifolia*
2.1.5. *Carpinus gigabracteatus*
2.2.6. *Carpinus hebestroma*
2.2.7. *Carpinus henryana*
2.2.8. *Carpinus hupeana*
2.2.9. *Carpinus insularis*
2.2.10. *Carpinus kawakamii*
2.2.11. *Carpinus kweichowensis*
2.1.12. *Carpinus mengshanensis*
2.2.13. *Carpinus microphylla*
2.2.14. *Carpinus minutiserrata*
2.2.15. *Carpinus mollicoma*
2.2.16. *Carpinus monbeigiana*
2.2.17. *Carpinus oblongifolia*
2.2.18. *Carpinus omeiensis*
2.2.19. *Carpinus polyneura*
2.2.20. *Carpinus pubescens*
2.2.21. *Carpinus purpurinervis*
2.2.22. *Carpinus putoensis*
2.2.23. *Carpinus rupestris*
2.2.24. *Carpinus shensiensis*
2.2.25. *Carpinus stipulata*
2.2.26. *Carpinus sungpanensis*
2.2.27. *Carpinus tibetana*
2.2.28. *Carpinus tsaiana*
2.2.29. *Carpinus tsunyihensis*
2.2.30. *Carpinus turczaninowii*
