# Supplementary material for: Delimiting 33 Carpinus (Betulaceae) species with a further phylogenetic inference
Source: AoB Plants. 2022 Feb 21;14(3):plac006. doi: 10.1093/aobpla/plac006 (PMC9162125; doi:10.1093/aobpla/plac006)
Supplement: plac006_suppl_Supplementary_Tables [file plac006_suppl_supplementary_tables.docx]

| Table S1. The collection information of 191 individuals in *Carpinus* and the haplotypes of all individuals in this study | | | | | | | | |
| --- | --- | --- | --- | --- | --- | --- | --- | --- |
| **Species** | **GenBank ID** | **Continent** | **Code** | **Location** | **Longitude** | **Latitude** | **Altitude** | **Haplotype** |
| *C. japonica** | AF432035 | Eastern Asia | — | — | — | — | — | R1 |
| *C. japonica** | FJ011718 | Eastern Asia | — | — | — | — | — | R1 |
| *C. japonica** | FJ011717 | Eastern Asia | — | — | — | — | — | R1 |
| *C. japonica** | FJ011716 | Eastern Asia | — | — | — | — | — | R1 |
| *C. japonica** | AJ783635 | Eastern Asia | — | — | — | — | — | R1 |
| *C. fangiana* | MW929069 | Eastern Asia | EM | Emei, Emei moutain, Sichuan | 103°24.000' | 29°34.000' | 1780 | R2 |
| *C. fangiana* | MW929068 | Eastern Asia | EM | Emei, Emei moutain, Sichuan | 103°24.000' | 29°34.000' | 1780 | R2 |
| *C. cordata* | MW929073 | Eastern Asia | LN | Tianmu moutain, Linan, Zhejiang | 119°26.100′ | 30°20.533′ | 1220 | R3 |
| *C. cordata* | MW929072 | Eastern Asia | LN | Tianmu moutain, Linan, Zhejiang | 119°26.100′ | 30°20.533′ | 1220 | R3 |
| *C. cordata* | MW929076 | Eastern Asia | ST | Huping moutain, Shimen, Hunan | 110°47.458′ | 30°03.226′ | 1655 | R3 |
| *C. cordata* | MW929075 | Eastern Asia | ST | Huping moutain, Shimen, Hunan | 110°47.458′ | 30°03.226′ | 1655 | R3 |
| *C. cordata* | MW929074 | Eastern Asia | ST | Huping moutain, Shimen, Hunan | 110°47.458′ | 30°03.226′ | 1655 | R3 |
| *C. cordata* | MW929071 | Eastern Asia | WF | Wantan, Wufeng, Hubei | 110°23.921' | 30°03.720' | 1362 | R3 |
| *C. cordata* | MW929070 | Eastern Asia | WF | Wantan, Wufeng, Hubei | 110°23.921' | 30°03.720' | 1362 | R3 |
| C. rankanensis* | FJ011728 | Eastern Asia | — | — | — | — | — | R4 |
| *C. rankanensis** | FJ011727 | Eastern Asia | — | — | — | — | — | R4 |
| *C. laxiflora** | AF432039 | Eastern Asia | — | — | — | — | — | R5 |
| *C. laxiflora** | AF432037 | Eastern Asia | — | — | — | — | — | R5 |
| *C. viminea* | MW928905 | Eastern Asia | CB | Dankou, Chengbu, Hunan | 110°11.750' | 26°23.900' | 1367 | R6 |
| *C. viminea* | MW928903 | Eastern Asia | CB | Dankou, Chengbu, Hunan | 110°11.750' | 26°23.900' | 1367 | R6 |
| *C. viminea* | MW928917 | Eastern Asia | CZ | Mangshan, Chenzhou, Hunan | 112°57.983′ | 24°58.917′ | 1680 | R6 |
| *C. viminea* | MW928916 | Eastern Asia | CZ | Mangshan, Chenzhou, Hunan | 112°57.983′ | 24°58.917′ | 1680 | R6 |
| *C. viminea* | MW928915 | Eastern Asia | CZ | Mangshan, Chenzhou, Hunan | 112°57.983′ | 24°58.917′ | 1680 | R6 |
| *C. viminea* | MW928914 | Eastern Asia | CZ | Mangshan, Chenzhou, Hunan | 112°57.983′ | 24°58.917′ | 1680 | R6 |
| **Species** | **GenBank ID** | **Continent** | **Code** | **Location** | **Longitude** | **Latitude** | **Altitude** | **Haplotype** |
| *C. viminea* | MW928913 | Eastern Asia | CZ | Mangshan, Chenzhou, Hunan | 112°57.983′ | 24°58.917′ | 1680 | R6 |
| *C. viminea* | MW928912 | Eastern Asia | CZ | Mangshan, Chenzhou, Hunan | 112°57.983′ | 24°58.917′ | 1680 | R6 |
| *C. viminea* | MW928911 | Eastern Asia | CZ | Mangshan, Chenzhou, Hunan | 112°57.983′ | 24°58.917′ | 1680 | R6 |
| *C. viminea* | MW928910 | Eastern Asia | CZ | Mangshan, Chenzhou, Hunan | 112°57.983′ | 24°58.917′ | 1680 | R6 |
| *C. viminea* | MW928909 | Eastern Asia | CZ | Mangshan, Chenzhou, Hunan | 112°57.983′ | 24°58.917′ | 1680 | R6 |
| *C. viminea* | MW928902 | Eastern Asia | DJ | Sanhe, Dejiang, Guizhou | 108°07.300' | 28°34.200' | 1076 | R6 |
| *C. viminea* | MW928901 | Eastern Asia | DJ | Sanhe, Dejiang, Guizhou | 108°07.300' | 28°34.200' | 1076 | R6 |
| *C. viminea* | MW928908 | Eastern Asia | HF | Mulinzi, Hefeng, Hubei | 110°12.360′ | 30°05.230′ | 1570 | R6 |
| *C. viminea* | MW928907 | Eastern Asia | HF | Mulinzi, Hefeng, Hubei | 110°12.360′ | 30°05.230′ | 1570 | R6 |
| *C. viminea* | MW928906 | Eastern Asia | HF | Mulinzi, Hefeng, Hubei | 110°12.360′ | 30°05.230′ | 1570 | R6 |
| *C. viminea* | MW928890 | Eastern Asia | HF | Mulinzi, Hefeng, Hubei | 110°12.360′ | 30°05.230′ | 1570 | R6 |
| *C. viminea* | MW928904 | Eastern Asia | LG | Hengxi, Langao, Shaanxi | 108°48.214' | 32°07.703' | 1165 | R6 |
| *C. viminea* | MW928920 | Eastern Asia | LL | Caoyuan, Longli, Guizhou | 106°55.000' | 26°19.000' | 1460 | R6 |
| *C. viminea* | MW928919 | Eastern Asia | LL | Caoyuan, Longli, Guizhou | 106°55.000' | 26°19.000' | 1460 | R6 |
| *C. viminea* | MW928918 | Eastern Asia | LL | Caoyuan, Longli, Guizhou | 106°55.000' | 26°19.000' | 1460 | R6 |
| *C. viminea* | MW928921 | Eastern Asia | ST | Huping moutain, Shimen, Hunan | 110°47.458′ | 30°03.226′ | 1655 | R6 |
| *C. viminea* | MW928899 | Eastern Asia | WF | Wantan, Wufeng, Hubei | 110°23.921' | 30°03.720' | 1362 | R6 |
| *C. viminea* | MW928898 | Eastern Asia | WF | Wantan, Wufeng, Hubei | 110°23.921' | 30°03.720' | 1362 | R6 |
| *C. viminea* | MW928891 | Eastern Asia | XF | Yanziba, Xianfeng, Hubei | 109°10.111' | 29°29.418' | 1103 | R6 |
| *C. viminea* | MW928900 | Eastern Asia | YS | Xiaoxi, Yongshun, Hunan | 110°16.000' | 28°48.000' | 602 | R6 |
| *C. viminea* | MW928897 | Eastern Asia | YS | Xiaoxi, Yongshun, Hunan | 110°16.000' | 28°48.000' | 602 | R6 |
| *C. viminea* | MW928896 | Eastern Asia | YS | Xiaoxi, Yongshun, Hunan | 110°16.000' | 28°48.000' | 602 | R6 |
| *C. viminea* | MW928895 | Eastern Asia | ZY | Jinding moutain, Zunyi, Guizhou | 106°48.199' | 27°44.414' | 1489 | R6 |
| *C. viminea* | MW928894 | Eastern Asia | ZY | Jinding moutain, Zunyi, Guizhou | 106°48.199' | 27°44.414' | 1489 | R6 |
| *C. viminea* | MW928893 | Eastern Asia | ZY | Jinding moutain, Zunyi, Guizhou | 106°48.199' | 27°44.414' | 1489 | R6 |
| **Species** | **GenBank ID** | **Continent** | **Code** | **Location** | **Longitude** | **Latitude** | **Altitude** | **Haplotype** |
| *C. viminea* | MW928892 | Eastern Asia | ZY | Jinding moutain, Zunyi, Guizhou | 106°48.199' | 27°44.414' | 1489 | R6 |
| *C.caroliniana** | AF432028 | North America | — | — | — | — | — | R7 |
| *C.caroliniana** | FJ011710 | North America | — | — | — | — | — | R7 |
| *C.caroliniana** | AJ783634 | North America | — | — | — | — | — | R8 |
| *C.caroliniana** | FJ011709 | North America | — | — | — | — | — | R8 |
| *C.caroliniana** | FJ011708 | North America | — | — | — | — | — | R8 |
| *C. betulus** | FJ011711 | Europea | — | — | — | — | — | R9 |
| *C. betulus** | MN808612 | Europea | — | — | — | — | — | R9 |
| *C. betulus** | MN808611 | Europea | — | — | — | — | — | R9 |
| *C. betulus** | MN808608 | Europea | — | — | — | — | — | R9 |
| *C. betulus** | MN808607 | Europea | — | — | — | — | — | R9 |
| *C. betulus** | MN808606 | Europea | — | — | — | — | — | R9 |
| *C. langaoensis* | MW929026 | Eastern Asia | LG | Hengxi, Langao, Shaanxi | 108°48.214' | 32°07.699' | 1165 | R10 |
| *C. langaoensis* | MW929025 | Eastern Asia | LG | Hengxi, Langao, Shaanxi | 108°48.214' | 32°07.700' | 1165 | R10 |
| *C. langaoensis* | MW929024 | Eastern Asia | LG | Hengxi, Langao, Shaanxi | 108°48.214' | 32°07.701' | 1165 | R10 |
| *C. langaoensis* | MW929023 | Eastern Asia | LG | Hengxi, Langao, Shaanxi | 108°48.214' | 32°07.702' | 1165 | R10 |
| *C. langaoensis* | MW929022 | Eastern Asia | LG | Hengxi, Langao, Shaanxi | 108°48.214' | 32°07.703' | 1165 | R10 |
| *C. mianningensis** | KX946971 | Eastern Asia | — | — | — | — | — | R11 |
| *C. mianningensis** | KX946972 | Eastern Asia | — | — | — | — | — | R11 |
| *C. putoensis* | OK560470 | Eastern Asia | PT | Putuo moutain, Zhoushan, Zhejiang | 122.396115 | 30.01692 | 250 | R12 |
| *C. putoensis* | OK560471 | Eastern Asia | PT | Putuo moutain, Zhoushan, Zhejiang | 122.396115 | 30.01692 | 250 | R12 |
| *C. putoensis* | OK560472 | Eastern Asia | PT | Putuo moutain, Zhoushan, Zhejiang | 122.396115 | 30.01692 | 250 | R12 |
| *C. putoensis* | OK560473 | Eastern Asia | PT | Putuo moutain, Zhoushan, Zhejiang | 122.396115 | 30.01692 | 250 | R12 |
| *C. putoensis* | OK560474 | Eastern Asia | PT | Putuo moutain, Zhoushan, Zhejiang | 122.396115 | 30.01692 | 250 | R12 |
| *C. putoensis* | OK560475 | Eastern Asia | PT | Putuo moutain, Zhoushan, Zhejiang | 122.396115 | 30.01692 | 250 | R12 |
| **Species** | **GenBank ID** | **Continent** | **Code** | **Location** | **Longitude** | **Latitude** | **Altitude** | **Haplotype** |
| *C. putoensis* | OK560476 | Eastern Asia | PT | Putuo moutain, Zhoushan, Zhejiang | 122.396115 | 30.01692 | 250 | R12 |
| *C. putoensis* | OK560477 | Eastern Asia | PT | Putuo moutain, Zhoushan, Zhejiang | 122.396115 | 30.01692 | 250 | R12 |
| *C. putoensis* | OK560478 | Eastern Asia | PT | Putuo moutain, Zhoushan, Zhejiang | 122.396115 | 30.01692 | 250 | R12 |
| *C. putoensis* | OK560479 | Eastern Asia | PT | Putuo moutain, Zhoushan, Zhejiang | 122.396115 | 30.01692 | 250 | R12 |
| *C. putoensis* | OK560480 | Eastern Asia | PT | Putuo moutain, Zhoushan, Zhejiang | 122.396115 | 30.01692 | 250 | R12 |
| *C. tschonoskii* | MW928934 | Eastern Asia | ZJ | Tianmen moutain, Zhangjiajie, Hunan | 110°28.617′ | 29°04.203′ | 1451 | R13 |
| *C. tschonoskii* | MW928933 | Eastern Asia | ZJ | Tianmen moutain, Zhangjiajie, Hunan | 110°28.617′ | 29°04.203′ | 1451 | R13 |
| *C. tschonoskii* | MW928932 | Eastern Asia | ZJ | Tianmen moutain, Zhangjiajie, Hunan | 110°28.617′ | 29°04.203′ | 1451 | R13 |
| *C. tschonoskii* | MW928960 | Eastern Asia | HF | Mulinzi, Hefeng, Hubei | 110°12.360′ | 30°05.230′ | 1570 | R14 |
| *C. tschonoskii* | MW928959 | Eastern Asia | HF | Mulinzi, Hefeng, Hubei | 110°12.360′ | 30°05.230′ | 1570 | R14 |
| *C. tschonoskii* | MW928958 | Eastern Asia | HF | Mulinzi, Hefeng, Hubei | 110°12.360′ | 30°05.230′ | 1570 | R14 |
| *C. tschonoskii* | MW928957 | Eastern Asia | HF | Mulinzi, Hefeng, Hubei | 110°12.360′ | 30°05.230′ | 1570 | R14 |
| *C. tschonoskii* | MW928956 | Eastern Asia | HF | Mulinzi, Hefeng, Hubei | 110°12.360′ | 30°05.230′ | 1570 | R14 |
| *C. tschonoskii* | MW928955 | Eastern Asia | HF | Mulinzi, Hefeng, Hubei | 110°12.360′ | 30°05.230′ | 1570 | R14 |
| *C. tschonoskii* | MW928954 | Eastern Asia | HF | Mulinzi, Hefeng, Hubei | 110°12.360′ | 30°05.230′ | 1570 | R14 |
| *C. tschonoskii* | MW928953 | Eastern Asia | HF | Mulinzi, Hefeng, Hubei | 110°12.360′ | 30°05.230′ | 1570 | R14 |
| *C. tschonoskii* | MW928952 | Eastern Asia | HF | Mulinzi, Hefeng, Hubei | 110°12.360′ | 30°05.230′ | 1570 | R14 |
| *C. tschonoskii* | MW928951 | Eastern Asia | HF | Mulinzi, Hefeng, Hubei | 110°12.360′ | 30°05.230′ | 1570 | R14 |
| *C. tschonoskii* | MW928950 | Eastern Asia | HF | Mulinzi, Hefeng, Hubei | 110°12.360′ | 30°05.230′ | 1570 | R14 |
| *C. tschonoskii* | MW928949 | Eastern Asia | HF | Mulinzi, Hefeng, Hubei | 110°12.360′ | 30°05.230′ | 1570 | R14 |
| *C. tschonoskii* | MW928948 | Eastern Asia | HF | Mulinzi, Hefeng, Hubei | 110°12.360′ | 30°05.230′ | 1570 | R14 |
| *C. tschonoskii* | MW928947 | Eastern Asia | HF | Mulinzi, Hefeng, Hubei | 110°12.360′ | 30°05.230′ | 1570 | R14 |
| *C. tschonoskii* | MW928946 | Eastern Asia | HF | Mulinzi, Hefeng, Hubei | 110°12.360′ | 30°05.230′ | 1570 | R14 |
| *C. tschonoskii* | MW928945 | Eastern Asia | HF | Mulinzi, Hefeng, Hubei | 110°12.360′ | 30°05.230′ | 1570 | R14 |
| *C. tschonoskii* | MW928944 | Eastern Asia | HF | Mulinzi, Hefeng, Hubei | 110°12.360′ | 30°05.230′ | 1570 | R14 |
| **Species** | **GenBank ID** | **Continent** | **Code** | **Location** | **Longitude** | **Latitude** | **Altitude** | **Haplotype** |
| *C. tschonoskii* | MW928943 | Eastern Asia | HF | Mulinzi, Hefeng, Hubei | 110°12.360′ | 30°05.230′ | 1570 | R14 |
| *C. tschonoskii* | MW928942 | Eastern Asia | HF | Mulinzi, Hefeng, Hubei | 110°12.360′ | 30°05.230′ | 1570 | R14 |
| *C. tschonoskii* | MW928941 | Eastern Asia | HF | Mulinzi, Hefeng, Hubei | 110°12.360′ | 30°05.230′ | 1570 | R14 |
| *C. tschonoskii* | MW928940 | Eastern Asia | HF | Mulinzi, Hefeng, Hubei | 110°12.360′ | 30°05.230′ | 1570 | R14 |
| *C. tschonoskii* | MW928939 | Eastern Asia | HF | Mulinzi, Hefeng, Hubei | 110°12.360′ | 30°05.230′ | 1570 | R14 |
| *C. tschonoskii* | MW928938 | Eastern Asia | HF | Mulinzi, Hefeng, Hubei | 110°12.360′ | 30°05.230′ | 1570 | R14 |
| *C. tschonoskii* | MW928937 | Eastern Asia | HF | Mulinzi, Hefeng, Hubei | 110°12.360′ | 30°05.230′ | 1570 | R14 |
| *C. tschonoskii* | MW928936 | Eastern Asia | HF | Mulinzi, Hefeng, Hubei | 110°12.360′ | 30°05.230′ | 1570 | R14 |
| *C. tschonoskii* | MW928935 | Eastern Asia | HF | Mulinzi, Hefeng, Hubei | 110°12.360′ | 30°05.230′ | 1570 | R14 |
| *C. tschonoskii* | MW928931 | Eastern Asia | HF | Mulinzi, Hefeng, Hubei | 110°12.360′ | 30°05.230′ | 1570 | R14 |
| *C. tschonoskii* | MW928930 | Eastern Asia | HF | Mulinzi, Hefeng, Hubei | 110°12.360′ | 30°05.230′ | 1570 | R14 |
| *C. tschonoskii* | MW928929 | Eastern Asia | HF | Mulinzi, Hefeng, Hubei | 110°12.360′ | 30°05.230′ | 1570 | R14 |
| *C. tschonoskii* | MW928928 | Eastern Asia | HF | Mulinzi, Hefeng, Hubei | 110°12.360′ | 30°05.230′ | 1570 | R14 |
| *C. tschonoskii* | MW928927 | Eastern Asia | HF | Mulinzi, Hefeng, Hubei | 110°12.360′ | 30°05.230′ | 1570 | R14 |
| *C. tschonoskii* | MW928926 | Eastern Asia | HF | Mulinzi, Hefeng, Hubei | 110°12.360′ | 30°05.230′ | 1570 | R14 |
| *C. tschonoskii* | MW928925 | Eastern Asia | HF | Mulinzi, Hefeng, Hubei | 110°12.360′ | 30°05.230′ | 1570 | R14 |
| *C. tientaiensis** | JF796534 | Eastern Asia | — | — | — | — | — | R15 |
| *C. tientaiensis** | KX946976 | Eastern Asia | — | — | — | — | — | R15 |
| *C. tientaiensis** | KX946975 | Eastern Asia | — | — | — | — | — | R15 |
| *C. fargesiana* | MW929060 | Eastern Asia | HX | Taibai moutain, Huxian, Shaanxi | 107°52.297' | 34°00.403' | 1896 | R16 |
| *C. fargesiana* | MW929059 | Eastern Asia | HX | Taibai moutain, Huxian, Shaanxi | 107°52.297' | 34°00.403' | 1896 | R16 |
| *C. fargesiana* | MW929067 | Eastern Asia | LX | Bipenggou, Lixian, Sichuan | 102°59.000' | 31°24.000' | 2380 | R16 |
| *C. fargesiana* | MW929066 | Eastern Asia | LX | Bipenggou, Lixian, Sichuan | 102°59.000' | 31°24.000' | 2380 | R16 |
| *C. fargesiana* | MW929065 | Eastern Asia | LX | Bipenggou, Lixian, Sichuan | 102°59.000' | 31°24.000' | 2380 | R16 |
| *C. fargesiana* | MW929064 | Eastern Asia | LX | Bipenggou, Lixian, Sichuan | 102°59.000' | 31°24.000' | 2380 | R16 |
| **Species** | **GenBank ID** | **Continent** | **Code** | **Location** | **Longitude** | **Latitude** | **Altitude** | **Haplotype** |
| *C. fargesiana* | MW929063 | Eastern Asia | LX | Bipenggou, Lixian, Sichuan | 102°59.000' | 31°24.000' | 2380 | R16 |
| *C. fargesiana* | MW929062 | Eastern Asia | LX | Bipenggou, Lixian, Sichuan | 102°59.000' | 31°24.000' | 2380 | R16 |
| *C. fargesiana* | MW929061 | Eastern Asia | LX | Bipenggou, Lixian, Sichuan | 102°59.000' | 31°24.000' | 2380 | R16 |
| *C. henryana* | MW929058 | Eastern Asia | SO | Huping moutain, Shimen, Hunan | 107°52.297' | 30°06.283′ | 1896 | R16 |
| *C. henryana* | MW929057 | Eastern Asia | SO | Huping moutain, Shimen, Hunan | 107°52.297' | 30°06.283′ | 1896 | R16 |
| *C. henryana* | MW929056 | Eastern Asia | SO | Huping moutain, Shimen, Hunan | 107°52.297' | 30°06.283′ | 1896 | R16 |
| *C. henryana* | MW929055 | Eastern Asia | SO | Huping moutain, Shimen, Hunan | 107°52.297' | 30°06.283′ | 1896 | R16 |
| *C. henryana* | MW929054 | Eastern Asia | SO | Huping moutain, Shimen, Hunan | 107°52.297' | 30°06.283′ | 1896 | R16 |
| *C. henryana* | MW929053 | Eastern Asia | SO | Huping moutain, Shimen, Hunan | 107°52.297' | 30°06.283′ | 1896 | R16 |
| *C. henryana* | MW929052 | Eastern Asia | SO | Huping moutain, Shimen, Hunan | 107°52.297' | 30°06.283′ | 1896 | R16 |
| *C. henryana* | MW929051 | Eastern Asia | SO | Huping moutain, Shimen, Hunan | 107°52.297' | 30°06.283′ | 1896 | R16 |
| C. henryana | MW929050 | Eastern Asia | WF | Wantan, Wufeng, Hubei | 110°23.921' | 30°03.720' | 1362 | R16 |
| *C. henryana* | MW929049 | Eastern Asia | WF | Wantan, Wufeng, Hubei | 110°23.921' | 30°03.720' | 1362 | R16 |
| *C. hupeana* | MW929049 | Eastern Asia | ZJ | Tianmen moutain, Zhangjiajie, Hunan | 110°28.617′ | 29°04.203′ | 1451 | R16 |
| *C. hupeana* | MW929047 | Eastern Asia | ZJ | Tianmen moutain, Zhangjiajie, Hunan | 110°28.617′ | 29°04.203′ | 1451 | R16 |
| *C. hupeana* | MW929046 | Eastern Asia | ZJ | Tianmen moutain, Zhangjiajie, Hunan | 110°28.617′ | 29°04.203′ | 1451 | R16 |
| *C. hupeana* | MW929045 | Eastern Asia | ZJ | Tianmen moutain, Zhangjiajie, Hunan | 110°28.617′ | 29°04.203′ | 1451 | R16 |
| *C. hupeana* | MW929044 | Eastern Asia | ZJ | Tianmen moutain, Zhangjiajie, Hunan | 110°28.617′ | 29°04.203′ | 1451 | R16 |
| *C. hupeana* | MW929043 | Eastern Asia | ZJ | Tianmen moutain, Zhangjiajie, Hunan | 110°28.617′ | 29°04.203′ | 1451 | R16 |
| *C. hupeana* | MW929042 | Eastern Asia | ZJ | Tianmen moutain, Zhangjiajie, Hunan | 110°28.617′ | 29°04.203′ | 1451 | R16 |
| *C. hupeana* | MW929041 | Eastern Asia | ZJ | Tianmen moutain, Zhangjiajie, Hunan | 110°28.617′ | 29°04.203′ | 1451 | R16 |
| *C. hupeana* | MW929040 | Eastern Asia | ZJ | Tianmen moutain, Zhangjiajie, Hunan | 110°28.617′ | 29°04.203′ | 1451 | R16 |
| *C. hupeana* | MW929039 | Eastern Asia | ZJ | Tianmen moutain, Zhangjiajie, Hunan | 110°28.617′ | 29°04.203′ | 1451 | R16 |
| *C. hupeana* | MW929038 | Eastern Asia | ZJ | Tianmen moutain, Zhangjiajie, Hunan | 110°28.617′ | 29°04.203′ | 1451 | R16 |
| *C. hupeana* | MW929037 | Eastern Asia | ZJ | Tianmen moutain, Zhangjiajie, Hunan | 110°28.617′ | 29°04.203′ | 1451 | R16 |
| **Species** | **GenBank ID** | **Continent** | **Code** | **Location** | **Longitude** | **Latitude** | **Altitude** | **Haplotype** |
| *C. hupeana* | MW929036 | Eastern Asia | ZJ | Tianmen moutain, Zhangjiajie, Hunan | 110°28.617′ | 29°04.203′ | 1451 | R16 |
| *C. hupeana* | MW929035 | Eastern Asia | ZJ | Tianmen moutain, Zhangjiajie, Hunan | 110°28.617′ | 29°04.203′ | 1451 | R16 |
| *C. hupeana* | MW929034 | Eastern Asia | ZJ | Tianmen moutain, Zhangjiajie, Hunan | 110°28.617′ | 29°04.203′ | 1451 | R16 |
| *C. hupeana* | MW929033 | Eastern Asia | ZJ | Tianmen moutain, Zhangjiajie, Hunan | 110°28.617′ | 29°04.203′ | 1451 | R16 |
| *C. hupeana* | MW929032 | Eastern Asia | ZJ | Tianmen moutain, Zhangjiajie, Hunan | 110°28.617′ | 29°04.203′ | 1451 | R16 |
| *C. hupeana* | MW929031 | Eastern Asia | ZJ | Tianmen moutain, Zhangjiajie, Hunan | 110°28.617′ | 29°04.203′ | 1451 | R16 |
| *C. hupeana* | MW929030 | Eastern Asia | ZJ | Tianmen moutain, Zhangjiajie, Hunan | 110°28.617′ | 29°04.203′ | 1451 | R16 |
| *C. hupeana* | MW929029 | Eastern Asia | ZJ | Tianmen moutain, Zhangjiajie, Hunan | 110°28.617′ | 29°04.203′ | 1451 | R16 |
| *C. pubescens* | MW929005 | Eastern Asia | AL | Dushan, Anlong, Guizhou | 110°28.617′ | 25°17.033′ | 1451 | R16 |
| *C. pubescens* | MW929000 | Eastern Asia | AL | Dushan, Anlong, Guizhou | 110°28.617′ | 25°17.033′ | 1451 | R16 |
| *C. pubescens* | MW929004 | Eastern Asia | AL | Dushan, Anlong, Guizhou | 110°28.617′ | 25°17.033′ | 1451 | R16 |
| *C. pubescens* | MW929003 | Eastern Asia | NP | Nonghua, Napo, Guangxi | 105°36.000′ | 25°17.000′ | 1260 | R16 |
| *C. pubescens* | MW929002 | Eastern Asia | XC | Xisha, Xichou, Yunnan | 110°28.617′ | 23°25.900′ | 1451 | R16 |
| *C. pubescens* | MW929001 | Eastern Asia | XC | Xisha, Xichou, Yunnan | 110°28.617′ | 23°25.900′ | 1451 | R16 |
| *C. purpurinervis* | MW928998 | Eastern Asia | AL | Dushan, Anlong, Guizhou | 110°28.617′ | 25°17.033′ | 1451 | R16 |
| *C. purpurinervis* | MW928997 | Eastern Asia | AL | Dushan, Anlong, Guizhou | 110°28.617′ | 25°17.033′ | 1451 | R16 |
| *C. purpurinervis* | MW928996 | Eastern Asia | AL | Dushan, Anlong, Guizhou | 110°28.617′ | 25°17.033′ | 1451 | R16 |
| *C. purpurinervis* | MW928995 | Eastern Asia | AL | Dushan, Anlong, Guizhou | 110°28.617′ | 25°17.033′ | 1451 | R16 |
| *C. purpurinervis* | MW928999 | Eastern Asia | LT | Weng'ang, Libo, Guizhou | 104°40.267′ | 25°17.500′ | 1660 | R16 |
| *C. shensiensis* | MW928992 | Eastern Asia | XA | Nanwutai, Xi'an, Shaaxi | 108°58.777′ | 33°59.429′ | 1652 | R16 |
| *C. shensiensis* | MW928991 | Eastern Asia | XA | Nanwutai, Xi'an, Shaaxi | 108°58.777′ | 33°59.429′ | 1652 | R16 |
| *C. shensiensis* | MW928990 | Eastern Asia | XA | Nanwutai, Xi'an, Shaaxi | 108°58.777′ | 33°59.429′ | 1652 | R16 |
| *C. stipulata* | MW928989 | Eastern Asia | SO | Huping moutain, Shimen, Hunan | 107°52.297' | 30°06.283′ | 1896 | R16 |
| *C. stipulata* | MW928988 | Eastern Asia | SO | Huping moutain, Shimen, Hunan | 107°52.297' | 30°06.283′ | 1896 | R16 |
| *C. stipulata* | MW928987 | Eastern Asia | SO | Huping moutain, Shimen, Hunan | 107°52.297' | 30°06.283′ | 1896 | R16 |
| **Species** | **GenBank ID** | **Continent** | **Code** | **Location** | **Longitude** | **Latitude** | **Altitude** | **Haplotype** |
| *C. sungpanensis* | MW928976 | Eastern Asia | LX | Bipenggou, Lixian, Sichuan | 102°59.000' | 31°24.000' | 2380 | R16 |
| *C. sungpanensis* | MW928975 | Eastern Asia | LX | Bipenggou, Lixian, Sichuan | 102°59.000' | 31°24.000' | 2380 | R16 |
| *C. sungpanensis* | MW928974 | Eastern Asia | LX | Bipenggou, Lixian, Sichuan | 102°59.000' | 31°24.000' | 2380 | R16 |
| *C. sungpanensis* | MW928973 | Eastern Asia | LX | Bipenggou, Lixian, Sichuan | 102°59.000' | 31°24.000' | 2380 | R16 |
| *C. sungpanensis* | MW928972 | Eastern Asia | LX | Bipenggou, Lixian, Sichuan | 102°59.000' | 31°24.000' | 2380 | R16 |
| *C. sungpanensis* | MW928986 | Eastern Asia | SO | Huping moutain, Shimen, Hunan | 107°52.297' | 30°06.283′ | 1896 | R16 |
| *C. sungpanensis* | MW928985 | Eastern Asia | SO | Huping moutain, Shimen, Hunan | 107°52.297' | 30°06.283′ | 1896 | R16 |
| *C. sungpanensis* | MW928984 | Eastern Asia | SO | Huping moutain, Shimen, Hunan | 107°52.297' | 30°06.283′ | 1896 | R16 |
| *C. sungpanensis* | MW928983 | Eastern Asia | SO | Huping moutain, Shimen, Hunan | 107°52.297' | 30°06.283′ | 1896 | R16 |
| *C. sungpanensis* | MW928982 | Eastern Asia | SO | Huping moutain, Shimen, Hunan | 107°52.297' | 30°06.283′ | 1896 | R16 |
| *C. sungpanensis* | MW928981 | Eastern Asia | SO | Huping moutain, Shimen, Hunan | 107°52.297' | 30°06.283′ | 1896 | R16 |
| *C. sungpanensis* | MW928980 | Eastern Asia | SO | Huping moutain, Shimen, Hunan | 107°52.297' | 30°06.283′ | 1896 | R16 |
| *C. sungpanensis* | MW928979 | Eastern Asia | SO | Huping moutain, Shimen, Hunan | 107°52.297' | 30°06.283′ | 1896 | R16 |
| *C. sungpanensis* | MW928978 | Eastern Asia | SO | Huping moutain, Shimen, Hunan | 107°52.297' | 30°06.283′ | 1896 | R16 |
| *C. sungpanensis* | MW928977 | Eastern Asia | SO | Huping moutain, Shimen, Hunan | 107°52.297' | 30°06.283′ | 1896 | R16 |
| *C. turczaninowii* | MW928924 | Eastern Asia | LO | Yawu moutain, Lingbao, Shaanxi | 105°36.383′ | 34°27.764' | 1120 | R16 |
| *C. turczaninowii* | MW928923 | Eastern Asia | LO | Yawu moutain, Lingbao, Shaanxi | 105°36.383′ | 34°27.764' | 1120 | R16 |
| *C. turczaninowii* | MW928922 | Eastern Asia | LO | Yawu moutain, Lingbao, Shaanxi | 105°36.383′ | 34°27.764' | 1120 | R16 |
| *C. hupeana* | MW929048 | Eastern Asia | WF | Wantan, Wufeng, Hunan | 110°23.921' | 30°03.720' | 1362 | R17 |
| *C. hupeana* | MW929028 | Eastern Asia | YS | Xiaoxi, Yongshun, Hunan | 110°16.000' | 28°48.000' | 602 | R17 |
| *C. chuniana* | MW929080 | Eastern Asia | RY | Wuzhi moutain, Ruyuan, Guangdong | 108°58.777′ | 24°55.283′ | 1652 | R18 |
| *C. chuniana* | MW929079 | Eastern Asia | RY | Wuzhi moutain, Ruyuan, Guangdong | 108°58.777′ | 24°55.283′ | 1652 | R18 |
| *C. chuniana* | MW929078 | Eastern Asia | RY | Wuzhi moutain, Ruyuan, Guangdong | 108°58.777′ | 24°55.283′ | 1652 | R18 |
| *C. chuniana* | MW929077 | Eastern Asia | XC | Xisha, Xichou, Yunnan | 110°28.617′ | 23°25.900′ | 1451 | R18 |
| *C. tsaiana* | MW928967 | Eastern Asia | AL | Dushan, Anlong, Guizhou | 110°28.617′ | 25°17.033′ | 1451 | R18 |
| **Species** | **GenBank ID** | **Continent** | **Code** | **Location** | **Longitude** | **Latitude** | **Altitude** | **Haplotype** |
| *C. tsaiana* | MW928966 | Eastern Asia | AL | Dushan, Anlong, Guizhou | 110°28.617′ | 25°17.033′ | 1451 | R18 |
| *C. tsaiana* | MW928965 | Eastern Asia | AL | Dushan, Anlong, Guizhou | 110°28.617′ | 25°17.033′ | 1451 | R18 |
| *C. tsaiana* | MW928968 | Eastern Asia | LT | Weng'ang, Libo, Guizhou | 104°40.267′ | 25°17.500′ | 1660 | R18 |
| *C. tsaiana* | MW928971 | Eastern Asia | LY | Tiankeng, Leye, Guangxi | 106°30.681' | 24°49.504' | 1460 | R18 |
| *C. tsaiana* | MW928970 | Eastern Asia | LY | Tiankeng, Leye, Guangxi | 106°30.681' | 24°49.504' | 1460 | R18 |
| *C. tsaiana* | MW928969 | Eastern Asia | LY | Tiankeng, Leye, Guangxi | 106°30.681' | 24°49.504' | 1460 | R18 |
| *C. tsaiana* | MW928964 | Eastern Asia | NP | Nonghua, Napo, Guangxi | 105°36.000′ | 25°17.000′ | 1260 | R18 |
| *C. tsaiana* | MW928963 | Eastern Asia | NP | Nonghua, Napo, Guangxi | 105°36.000′ | 25°17.000′ | 1260 | R18 |
| *C. tsaiana* | MW928962 | Eastern Asia | NP | Nonghua, Napo, Guangxi | 105°36.000′ | 25°17.000′ | 1260 | R18 |
| *C. tsaiana* | MW928961 | Eastern Asia | NP | Nonghua, Napo, Guangxi | 105°36.000′ | 25°17.000′ | 1260 | R18 |
| *C. monbeigiana* | MW929017 | Eastern Asia | KM | Xi moutain, Kunming, Yunnan | 102°38.368' | 24°58.137' | 2355 | R19 |
| *C. monbeigiana* | MW929016 | Eastern Asia | KM | Xi moutain, Kunming, Yunnan | 102°38.368' | 24°58.137' | 2355 | R19 |
| *C. kawakamii** | FJ011720 | Eastern Asia | — | — | — | — | — | R20 |
| *C. kawakamii** | FJ011719 | Eastern Asia | — | — | — | — | — | R20 |
| *C. tibetana** | KY436145 | Eastern Asia | — | — | — | — | — | R21 |
| *C. tibetana** | KY436146 | Eastern Asia | — | — | — | — | — | R21 |
| *C. tibetana** | KY436147 | Eastern Asia | — | — | — | — | — | R21 |
| *C.orientalis** | FJ011725 | European | — | — | — | — | — | R21 |
| *C. orientalis** | AF432049 | European | — | — | — | — | — | R22 |
| *C. orientalis** | FJ011724 | European | — | — | — | — | — | R22 |
| *C. londoniana* | MW929021 | Eastern Asia | YS | Xiaoxi, Yongshun, Hunan | 110°16.000' | 28°48.000' | 602 | R23 |
| *C. londoniana* | MW929020 | Eastern Asia | YS | Xiaoxi, Yongshun, Hunan | 110°16.000' | 28°48.000' | 602 | R23 |
| *C. polyneura* | MW929013 | Eastern Asia | DJ | Sanhe, Dejiang, Guizhou | 108°07.300' | 28°34.200' | 1076 | R23 |
| *C. polyneura* | MW929012 | Eastern Asia | DJ | Sanhe, Dejiang, Guizhou | 108°07.300' | 28°34.200' | 1076 | R23 |
| *C. polyneura* | MW929006 | Eastern Asia | HF | Mulinzi, Hefeng, Hubei | 110°12.360′ | 30°05.230′ | 1570 | R24 |
| **Species** | **GenBank ID** | **Continent** | **Code** | **Location** | **Longitude** | **Latitude** | **Altitude** | **Haplotype** |
| *C. polyneura* | MW929009 | Eastern Asia | XF | Yanziba, Xianfeng, Hubei | 109°10.111' | 29°29.418' | 1103 | R24 |
| *C. polyneura* | MW929011 | Eastern Asia | WF | Wantan, Wufeng, Hubei | 110°23.921' | 30°03.720' | 1362 | R25 |
| *C. polyneura* | MW929010 | Eastern Asia | WF | Wantan, Wufeng, Hubei | 110°23.921' | 30°03.720' | 1362 | R25 |
| *C. mollicoma* | MW929019 | Eastern Asia | XC | Xisha, Xichou, Yunnan | 110°28.617′ | 23°25.900′ | 1451 | R26 |
| *C. mollicoma* | MW929018 | Eastern Asia | XC | Xisha, Xichou, Yunnan | 110°28.617′ | 23°25.900′ | 1451 | R26 |
| *C. omeiensis* | MW929015 | Eastern Asia | TL | Laoshan, Tianlin, Guangxi | 106°20.605′ | 24°22.843′ | 1023 | R26 |
| *C. omeiensis* | MW929014 | Eastern Asia | TL | Laoshan, Tianlin, Guangxi | 106°20.605′ | 24°22.843′ | 1023 | R26 |
| *C. polyneura* | MW929008 | Eastern Asia | XC | Xisha, Xichou, Yunan | 110°28.617′ | 23°25.900′ | 1451 | R26 |
| *C. polyneura* | MW929007 | Eastern Asia | XC | Xisha, Xichou, Yunan | 110°28.617′ | 23°25.900′ | 1451 | R26 |
| *C. rupestris* | MW928994 | Eastern Asia | XC | Xisha, Xichou, Yunan | 110°28.617′ | 23°25.900′ | 1451 | R26 |
| *C. rupestris* | MW928993 | Eastern Asia | XC | Xisha, Xichou, Yunan | 110°28.617′ | 23°25.900′ | 1451 | R26 |

### “*” means the sequence downloaded from NCBI, and “—” means information missing.

| Table S2. All sequences downloaded from NCBI in this study | | | | |
| --- | --- | --- | --- | --- |
| **Species** | **GenBank ID** |  | **Species** | **GenBank ID** |
| *Corylus californica* | AF297343 |  | *Carpinus tibetana* | KY436147 |
| *Corylus heterophylla* | AF297350 |  | *Carpinus tientaiensis* | JF796534 |
| *Carpinus betulus* | FJ011711 |  | *Carpinus tientaiensis* | KX946976 |
| *Carpinus betulus* | MN808612 |  | *Carpinus tientaiensis* | KX946975 |
| *Carpinus betulus* | MN808611 |  | *Ostrya carpinifolia* | FJ011752 |
| Carpinus betulus | MN808608 |  | *Ostrya carpinifolia* | AF432059 |
| *Carpinus betulus* | MN808607 |  | *Ostrya japonica* | AF432060 |
| Carpinus betulus | MN808606 |  | *Ostrya japonica* | AJ783637 |
| *Carpinus caroliniana* | AF432028 |  | *Ostrya japonica* | FJ011754 |
| *Carpinus caroliniana* | FJ011710 |  | *Ostrya japonica* | KX305969 |
| *Carpinus caroliniana* | AJ783634 |  | *Ostrya japonica* | KX305971 |
| *Carpinus caroliniana* | FJ011709 |  | *Ostrya japonica* | KX305972 |
| *Carpinus caroliniana* | FJ011708 |  | *Ostrya japonica* | KX305970 |
| *Carpinus japonica* | AF432035 |  | *Ostrya knowltonii* | FJ011755 |
| *Carpinus japonica* | FJ011718 |  | *Ostrya knowltonii* | AF432061 |
| *Carpinus japonica* | FJ011717 |  | *Ostrya multinervis* | KX305966 |
| *Carpinus japonica* | FJ011716 |  | *Ostrya multinervis* | KX305965 |
| *Carpinus japonica* | AJ783635 |  | *Ostrya rehderiana* | AF432062 |
| *Carpinus kawakamii* | FJ011720 |  | *Ostrya rehderiana* | AF432063 |
| *Carpinus kawakamii* | FJ011719 |  | *Ostrya rehderiana* | KX305960 |
| *Carpinus laxiflora* | AF432039 |  | *Ostrya rehderiana* | KX305959 |
| *Carpinus laxiflora* | AF432037 |  | *Ostrya rehderiana* | FJ011756 |
| *Carpinus mianningensis* | KX946971 |  | *Ostrya trichocarpa* | KX305964 |
| *Carpinus mianningensis* | KX946972 |  | *Ostrya trichocarpa* | KX305961 |
| *Carpinus orientalis* | FJ011725 |  | *Ostrya trichocarpa* | KX305962 |
| *Carpinus orientalis* | AF432049 |  | *Ostrya trichocarpa* | KX305963 |
| *Carpinus orientalis* | FJ011724 |  | *Ostrya virginiana* | FJ011758 |
| Carpinus putoensis | AF432051 |  | *Ostrya virginiana* | FJ011757 |
| *Carpinus rankanensis* | FJ011728 |  | *Ostrya virginiana* | AF432064 |
| *Carpinus rankanensis* | FJ011727 |  | *Ostrya yunnanensis* | KX305967 |
| *Carpinus tibetana* | KY436145 |  | *Ostrya yunnanensis* | KX305968 |
| *Carpinus tibetana* | KY436146 |  |  |  |

| **Table S3. The haplotypes of all *Carpinus* samples in this study and their corresponding species** | | | | |
| --- | --- | --- | --- | --- |
| **Species** | **Haplotype (Number)** | **Continent** | **Location** | **Code** |
| C. betulus | R9(6) | Europea | — | — |
| *C. caroliniana* | R7(2),R8(3) | North America | — | — |
| *C. chuniana* | R18(4) | Eastern Asia | Xisha, Xichou, Yunnan | XC |
|  |  | Eastern Asia | Wuzhi moutain, Ruyuan, Guangdong | RY |
| *C. cordata* | R3(7) | Eastern Asia | Tianmu moutain, Linan, Zhejiang | LN |
|  |  | Eastern Asia | Wantan, Wufeng, Hubei | WF |
|  |  | Eastern Asia | Huping moutain, Shimen, Hunan | ST |
| *C. fangiana* | R2(2) | Eastern Asia | Emei, Emei moutain, Sichuan | EM |
| *C. fargesiana* | R16(9) | Eastern Asia | Taibai moutain, Huxian, Shaanxi | HX |
|  |  | Eastern Asia | Bipenggou, Lixian, Sichuan | LX |
| *C. henryana* | R16(10) | Eastern Asia | Wantan, Wufeng, Hubei | WF |
|  |  | Eastern Asia | Huping moutain, Shimen, Hunan | SO |
|  |  | Eastern Asia | Tianmen moutain, Zhangjiajie, Hunan | ZJ |
| *C. hupeana* | R16(20),R17(2) | Eastern Asia | Wantan, Wufeng, Hubei | WF |
|  |  | Eastern Asia | Xiaoxi, Yongshun, Hunan | YS |
| *C. japonica* | R1(5) | Eastern Asia | — | — |
| *C. kawakamii* | R20(2) | Eastern Asia | — | — |
| *C. langaoensis* | R6(1),R10(5) | Eastern Asia | Hengxi, Langao, Shaanxi | LG |
| *C. laxiflora* | R5(2) | Eastern Asia | — | — |
| *C. londoniana* | R23(2) | Eastern Asia | Xiaoxi, Yongshun, Hunan | YS |
| *C. mianningensis* | R11(2),R12(1) | Eastern Asia | — | — |
|  |  | Eastern Asia | Putuo moutain, Zhoushan, Zhejiang | PT |
| **Species** | **Haplotype (Number)** | **Continent** | **Location** | **Code** |
| *C. mollicoma* | R26(2) | Eastern Asia | Xisha, Xichou, Yunnan | XC |
| *C. monbeigiana* | R19(2) | Eastern Asia | Xi moutain, Kunming, Yunnan | KM |
| *C. omeiensis* | R26(2) | Eastern Asia | Laoshan, Tianlin, Guangxi | TL |
| *C. orientalis* | R21(1),R22(2) | European | — | — |
| *C. purpurinervis* | R16(4),R18(1) | Eastern Asia | Dushan, Anlong, Guizhou | AL |
|  |  | Eastern Asia | Weng'ang, Libo, Guizhou | LT |
| *C. putoensis* | R12(9) | Eastern Asia | Putuo moutain, Zhoushan, Zhejiang | PT |
| *C. rankanensis* | R5(2) | Eastern Asia | — | — |
| *C. rupestris* | R26(2) | Eastern Asia | Xisha, Xichou, Yunnan | XC |
| *C. shensiensis* | R16(3) | Eastern Asia | Nanwutai, Xi'an, Shaaxi | XA |
| *C. polyneura* | R23(2),R24(2),R25(2),R26(2) | Eastern Asia | Sanhe, Dejiang, Guizhou | DJ |
|  |  | Eastern Asia | Mulinzi, Hefeng, Hubei | HF |
|  |  | Eastern Asia | Yanziba, Xianfeng, Hubei | XF |
|  |  | Eastern Asia | Wantan, Wufeng, Hubei | WF |
|  |  | Eastern Asia | Xisha, Xichou, Yunnan | XC |
| *C. pubescens* | R16(6) | Eastern Asia | Nonghua, Napo, Guangxi | NP |
|  |  | Eastern Asia | Xisha, Xichou, Yunnan | XC |
|  |  | Eastern Asia | Dushan, Anlong, Guizhou | AL |
| *C. stipulata* | R16(3) | Eastern Asia | Huping moutain, Shimen, Hunan | SO |
| *C. sungpanensis* | R16(15) | Eastern Asia | Huping moutain, Shimen, Hunan | SO |
|  |  | Eastern Asia | Bipenggou, Lixian, Sichuan | LX |
| *C. tibetana* | R21(3) | Eastern Asia | — | — |
| *C. tschonoskii* | R12(2),R13(3),R14(33) | Eastern Asia | Tianmen moutain, Zhangjiajie, Hunan | ZJ |
| **Species** | **Haplotype (Number)** | **Continent** | **Location** | **Code** |
| *C. tschonoskii* | R12(2),R13(3),R14(33) | Eastern Asia | Mulinzi, Hefeng, Hubei | HF |
|  |  | Eastern Asia | Putuo moutain, Zhoushan, Zhejiang | PT |
| *C. tientaiensis* | R15(3) | Eastern Asia | — | — |
| *C. tsaiana* | R16(1),R18(10) | Eastern Asia | Weng'ang, Libo, Guizhou | LT |
|  |  | Eastern Asia | Dushan, Anlong, Guizhou | AL |
|  |  | Eastern Asia | Tiankeng, Leye, Guangxi | LY |
|  |  | Eastern Asia | Nonghua, Napo, Guangxi | NP |
| *C. turczaninowii* | R16(3) | Eastern Asia | Yawu moutain, Lingbao, Shaanxi | LO |
| C. viminea | R6(31) | Eastern Asia | Huping moutain, Shimen, Hunan | ST |
|  |  | Eastern Asia | Yanziba, Xianfeng, Hubei | XF |
|  |  | Eastern Asia | Wantan, Wufeng, Hubei | WF |
|  |  | Eastern Asia | Dankou, Chengbu, Hunan | CB |
|  |  | Eastern Asia | Sanhe, Dejiang, Guizhou | DJ |
|  |  | Eastern Asia | Caoyuan, Longli, Guizhou | LL |
|  |  | Eastern Asia | Xiaoxi, Yongshun, Hunan | YS |
|  |  | Eastern Asia | Jinding moutain, Zunyi, Guizhou | ZY |
|  |  | Eastern Asia | Mulinzi, Hefeng, Hubei | HF |
|  |  | Eastern Asia | Mangshan, Chenzhou, Hunan | CZ |
